# *Staphylococcus aureus* and *Escherichia hermanii* in Diabetes Patient

**DOI:** 10.3201/eid1007.030567

**Published:** 2004-07

**Authors:** Gabriel Adrian Popescu, Ioana Daha, Cristina Popescu, Elena Mitache

**Affiliations:** * Matei Bals Infectious Diseases Institute, Bucharest, Romania;; † Colentina Hospital, Bucharest, Romania;; ‡ Carol Davila University of Medicine, Bucharest, Romania

**Keywords:** *S. aureus*, *E. Hermanii*, *Staphylococcus aureus*, *Escherichia hermanii*

**To the Editor:** Polymicrobial invasive infections are infrequent, representing <10% of the invasive infections of known etiology (1). They are often correlated with a predisposing factor: immunodeficiency (e.g., diabetes mellitus, malignancies, extremes of age) or use of a central catheter. *Escherichia hermanii* is an extremely rare etiologic agent for invasive infections; only four cases were published from 1980 to 2002. We report the first case of double invasive infection by *E. hermanii* and *Staphylococcus aureus* and emphasize the importance of screening of all the septic foci for demonstrating a polymicrobial invasive infection.

In August 2000, a 54-year-old comatose man was admitted to our infectious diseases department with a 10-day history of fever. He had a medical history of vertebral arthrosis (lumbar laminectomy in 1989) and insulin-dependent diabetes mellitus. Six weeks before, he had received for 3 days gluteal injections with kebusone (an intramuscular nonsteriodal antiinflammatory drug [NSAID]) for acute lower back pain. Twenty-eight days after the first injection, a gluteal abscess developed, which was surgically drained, without perioperative antimicrobial therapy. Three days later, he became febrile, and pyrexia persisted despite local wound management and treatment with oxacillin, 4 g/day for 3 days; cefuroxime, 3 g/day, and gentamicin, 160 mg/day for another 7 days.

The patient became comatose and was transferred to our department. At that time, the physical examination showed fever (40.2°C), neck stiffness, Brudzinski sign, thoracic dullness, and bilateral crackling rales. The level of C-reactive protein was 123 mg/L. Renal failure was noted with a creatinine blood level of 312 mmol/L and uncontrolled diabetes with fasting glucose of 24.75 mmol/L. Computed tomographic (CT) scan of the brain did not show brain abscesses or tumors. Examination of the cerebrospinal fluid (CSF) indicated a protein level of 2.67 g/L, decreased glucose concentration of 0.55 mmol/L, and a leukocyte count of 2.3 x 10^9^/L with 96% neutrophils; no microbial pathogens were demonstrable under direct examination of CSF. Chest x-ray identified bronchopneumonia and bilateral pleural effusion. The pleural fluid analysis revealed a purulent exudate—protein, 4.5 g/L—containing 55% neutrophils. A urine specimen and three blood samples were obtained for cultures over the first 4 hours after admission. A bacterial invasive infection was considered and the antibiotic therapy was started with ceftriaxone, 2 g/day, and rifampin, 1,200 mg/day. Concomitantly, the patient received colloids to reestablish blood volume, intravenous dexamethasone, 6 mg four times daily, to diminish the cerebral edema; and fast-acting insulin to control hyperglycemia.

On day 3, the urine and CSF cultures were positive for *E. hermanii*, and the pleural fluid and all three blood cultures yielded methicillin-susceptible *S. aureus*. The *E. hermanii* strain produced a yellow pigment. The drug susceptibility was assessed by AtB Expression system (BioMerieux, Marcy l'Etoile, France). The *Staphylococcus* strain was susceptible to oxacillin, cotrimoxazole, tetracycline, and ciprofloxacin and resistant only to penicillin. *E. hermanii* is naturally resistant to aminopenicillins and carbenicillin; this strain was susceptible to third-generation cephalosporins, carbapenems, cotrimoxazole, and quinolones and resistant to aminoglycosides.

The patient's clinical central nervous system status improved, and he came out of the coma, but his temperature remained >37.5°C. He started to report lumbar pain. On day 5, the antimicrobial regimen was switched to meropenem. After 24 hours, the patient became apyretic, and glucose and creatinine levels were normal on day 8. However, on day 10, fever, inflammation of the right thumb, and intensified lower back pain developed. The abdominal CT and bone scintigraphy indicated abscess of the psoas, L4-L5 spondylitis, and thumb periostitis. Intravenous ciprofloxacin was added, 400 mg twice daily, and apyrexia occurred on day 14. On day 21, open surgery was performed, consisting of drainage of the psoas abscess and curettage of the L4-L5 disc. On day 30, clinical improvement and C-reactive protein level of 4.2 mg/L, led to a change to oral antimicrobial agents: cotrimoxazole 2 g/day and ciprofloxacin 1.5 g/day. This regimen was continued for 2 months while the patient was seen as an outpatient. The patient remained afebrile and inflammation-free for the entire 24-month followup period.

Polymicrobial invasive infections represent a major therapeutic problem. However, they are infrequent: only 3.2% of infectious endocarditis (2) cases and 6.27% of 2,188 community-acquired bacteremia cases (3) were polymicrobial. Polymicrobial infections and elevated bacteremia levels are more frequently associated with diabetes; 20%–35% of the skin and soft tissue infections in persons with diabetes are polymicrobial (4), and 15.7%–20% of the community-acquired bacteremia cases were registered in persons with diabetes (3,4). The probable entry site for *S. aureus* was cutaneous. Intramuscular NSAIDs are known to cause aseptic necrosis, predisposing the patient to staphylococcal abscesses. Hyperglycemia itself is a risk factor for soft-tissue infections. The role of perioperative antimicrobial therapy in preventing the dissemination of infection from a surgically drained abscess is controversial (5). *E. hermanii* usually produces wound or gastrointestinal tract infections; in our patient, *E. hermanii* probably originated from the skin or from the gastrointestinal tract (6–9). *E. hermanii* could be involved more frequently in polymicrobial invasive infections; of *E. hermanii* invasive infections noted in four published reports, two were polymicrobial (6,7).

Initial antimicrobial drug therapy was established empirically for probable staphylococcal meningitis. The ongoing fever and persistent metabolic disturbances led to an escalation of therapy. Some authors recommend carbapenems as the initial regimen against invasive methicillin-susceptible *S. aureus* infections with meningeal or bone involvement (10). The patient's lower back and the invasive staphylococcal infection urged us to consider septic bone involvement. The imaging studies confirmed the existence of vertebral osteomyelitis; ciprofloxacin was used based on its excellent bone diffusion and its in vitro activity on the two isolated strains. Surgical intervention eradicated of one of the septic foci and decreased risk for spinal cord injury; 27 (47%) of the 58 patients with spondylodiscitis who were treated surgically had a better outcome than the other patients with medical care only (11). Control of the infection allowed changing to an oral regimen after 1 month. We selected cotrimoxazole and ciprofloxacin for their in vitro effectiveness against the two pathogens. Although this agent is not usually used to treat bone infection, we used cotrimoxazole on the basis of evidence provided by several communications that indicated a superior efficiency to referential regimens (12).

In conclusion, the identification of all organisms involved in polymicrobial invasive infections may require cultures of specimens from all accessible septic foci. For *E. hermanii*, a role of "associated" pathogen in a polymicrobial invasive infection could be considered. Medical therapy alone could be insufficient, and the combined therapy allowed for a successful outcome in invasive infection with lumbar spondilodiscitis.
